# Genome-wide analysis of long non-coding RNAs in *Catalpa bungei* and their potential function in floral transition using high-throughput sequencing

**DOI:** 10.1186/s12863-018-0671-2

**Published:** 2018-09-20

**Authors:** Zhi Wang, Tianqing Zhu, Wenjun Ma, Nan Wang, Guanzheng Qu, Shougong Zhang, Junhui Wang

**Affiliations:** 1 State Key Laboratory of Tree Genetics and Breeding, Key Laboratory of Tree Breeding and Cultivation of State Forestry Administration, Research Institute of Forestry, Chinese Academy of Forestry, Haidian District, Dongxiaofu 1#, Beijing, 100091 People’s Republic of China; 2 State Key Laboratory of Tree Genetics and Breeding (Northeast Forestry University), 26 Hexing Road, Harbin, 150040 People’s Republic of China

**Keywords:** Long non-coding RNAs, Woody plant, Floral transition, RNA-sequencing, *Catalpa bungei*

## Abstract

**Background:**

Long non-coding RNAs (lncRNAs) have crucial roles in various biological regulatory processes. However, the study of lncRNAs is limited in woody plants. *Catalpa bungei* is a valuable ornamental tree with a long cultivation history in China, and a deeper understanding of the floral transition mechanism in *C. bungei* would be interesting from both economic and scientific perspectives.

**Results:**

In this study, we categorized *C. bungei* buds from early flowering (EF) and normal flowering (NF) varieties into three consecutive developmental stages. These buds were used to systematically study lncRNAs during floral transition using high-throughput sequencing to identify molecular regulatory networks. Quantitative real-time PCR was performed to study RNA expression changes in different stages. In total, 12,532 lncRNAs and 26,936 messenger RNAs (mRNAs) were detected. Moreover, 680 differentially expressed genes and 817 differentially expressed lncRNAs were detected during the initiation of floral transition. The results highlight the mRNAs and lncRNAs that may be involved in floral transition, as well as the many lncRNAs serving as microRNA precursors. We predicted the functions of lncRNAs by analysing the relationships between lncRNAs and mRNAs. Seven lncRNA-mRNA interaction pairs may participate in floral transition.

**Conclusions:**

This study is the first to identify lncRNAs and their potential functions in floral transition, providing a starting point for detailed determination of the functions of lncRNAs in *C. bungei*.

**Electronic supplementary material:**

The online version of this article (10.1186/s12863-018-0671-2) contains supplementary material, which is available to authorized users.

## Background

In the early 1990s, a new class of RNA was identified [1–3]; the RNAs in this class, known as non-coding RNAs (ncRNAs), are transcripts that lack protein-coding potential [4]. Based on product length, ncRNAs can be subdivided into two groups: small ncRNAs (< 200 nt), which are mainly microRNAs (miRNAs) and small RNAs (sRNAs), and long ncRNAs (lncRNAs; > 200 nt), which include long intronic ncRNAs and long intergenic ncRNAs [5]. lncRNAs can be divided into three forms based on their effects: miRNA precursors, natural antisense transcripts (NATs), and lncRNAs that bind with miRNAs to sequester the regulatory roles of miRNAs on their target genes.

High-throughput genomic technologies have advanced our understanding of lncRNAs in the last decade. However, most studies on lncRNAs have concentrated on animals, and few studies have been performed in plants [2, 5]. To date, lncRNAs have been studied in only some plant species, mostly vegetables or herbs, but also several woody plants [6–8].

In plants, lncRNAs are considered important in fertility [7], fruit ripening [9], DNA methylation [10, 11], flowering time [12, 13], and photomorphogenesis [14]. COLDAIR and COOLAIR are the most well studied lncRNAs in plants [12–16]; they participate in the repression of Flowering Locus C (FLC) during vernalization in *Arabidopsis* and are involved in flower transition [12]. However, few studies on lncRNAs related to floral transition in woody plants are available. *Catalpa bungei* (family: Bignoniaceae) is a valuable timber material also used in Chinese medicine. Furthermore, *C. bungei* is famous for its beautiful flowers and is an ancient ornamental woody plant widely distributed in the middle and western regions of China. *C. bungei* typically experiences its first floral transition in trees that are five years old or older. However, an early flowering (EF) variety undergoes flower transition in the first year after planting. Here, we used this EF variety to study the lncRNAs expressed during the transition from vegetative growth to reproductive growth in *C. bungei* with plants provided by the Henan Provincial Department of Forestry (http://www.hnly.gov.cn).

The Illumina Hi-Seq sequencing platform was used to further investigate the genes and lncRNAs involved in floral transition. In the present study, a comprehensive analysis of lncRNAs and mRNAs from EF and normal flowering (NF) varieties during three development periods was performed. A total of 680 differentially expressed genes (DEGs) and 817 differentially expressed lncRNAs (DELs) were identified during the initiation of floral transition. Further analysis indicated that lncRNAs show several distinctions from mRNAs. For example, numerous lncRNAs are precursors to miRNAs and many lncRNAs regulate protein-coding genes’ expression. This study provides fundamental information to aid future investigations of floral transition in *C. bungei*. This is the first study to identify and characterize the lncRNAs present during floral transition in woody plants.

## Methods

### Plant materials

*C. bungei* is a perennial tree that typically flowers after seven years. However, a natural EF variety of *C. bungei* that flowers after one year was found in Henan Province, China, and was used to create a new variety, “bairihua”. From February 28 to March 31, 2016, we collected the first round of axillary buds of EF and NF varieties every one to two days. The samples used for RNA extraction were washed with distilled water, frozen immediately in liquid nitrogen, and stored at − 80 °C. Samples for histological analysis were fixed in formalin: glacial acetic acid: 70% ethanol (5:5:90 vol.; FAA) solution under a vacuum for at least 24 h.

### Histological analysis

After FAA fixation, the samples were dehydrated according to the methods of a previous study [17–19], processed with three changes of 100% paraffin at 63 °C, and finally embedded. The paraffin-embedded material was cut into 10-mm-thick sections (RM2255 Fully Automated Rotary Microtome; Leica, Germany), and the sections were stained with Safranin O and fast green FCF (Sigma-Aldrich, USA) [17]. The slices were observed and photographed using a Nikon D3000 camera, a Leica M205 FA fluorescence stereo microscope, and a Leica DM 6000B fully automated upright microscope (Leica Microsystems GmbH, Wetzlar, Germany).

### Total RNA isolation, library construction, and Illumina transcriptome sequencing

Total RNA was isolated using the Spectrum Plant Total RNA Kit (Sigma-Aldrich, USA) following the manufacturer’s protocol. Total RNA quality was monitored by ultraviolet spectrophotometry (NanoDrop 8000 Spectrophotometer; Thermo Scientific, USA). First-stand complementary DNA (cDNA) was synthesized using the First Strand cDNA Synthesis Kit (TaKaRa, Japan) following the manufacturer’s instructions.

The EF and NF buds were grouped into three developmental periods according to the histological analysis. Three replicates were included for each period. Libraries were constructed using the second-generation TruSeq Stranded RNA Kit (Illumina Inc., San Diego, USA) following the manufacturer’s recommendations. In total, 18 cDNA libraries, which were sequenced using an Illumina Solexa sequencer, were constructed. To assess the quality of the RNA-sequencing (RNA-seq) data, each base in the reads was assigned a quality score (Q) with a Phred-like algorithm using SOAPnuke software (http://soap.genomics.org.cn/) [20].

### lncRNA identification

Transcripts were assembled and merged using Cufflinks software according to the software instructions [21]. The initial assembled transcripts were compared to known *C. bungei* transcripts using Cuffcompare software [22–24]. High-quality assemblies with lengths ≥200 bp were retrieved. Three prediction programmes, CPC (http://www.mybiosoftware.com/cpc-0-9r2-assess-protein-coding-potential-transcripts.html), txCdspredict (http://hgdownload.soe.ucsc.edu/admin/jksrc.zip), and CNCI (https://github.com/www-bioinfo-org/CNCI), were used to predict the protein-coding ability of the transcripts [22–24]. Score thresholds were set to distinguish lncRNA from mRNA (CPC threshold: ≥ 0 = mRNA, < 0 = lncRNA; txCdspredict threshold: ≥ 500 = mRNA, < 500 = lncRNA; CNCI threshold: ≥ 0 = mRNA, < 0 = lncRNA) [22]. Transcripts that could be aligned in the protein database Pfam were predicted to be mRNA, while those that could not be aligned in Pfam were predicted to be lncRNA [25]. Transcripts reported as lncRNAs by at least three of the four above prediction methods were identified as lncRNAs.

### lncRNAs predicted to be miRNA targets

DEL target genes were identified based on their *trans*-regulatory effects using sequence complementary analysis, as described previously. To explore whether lncRNAs function as miRNA decoys, the lncRNAs were submitted to the psRNATarget server (http://plantgrn.noble.org/psRNATarget/) with an expectation value < 3. lncRNAs containing no more than four mismatches and G/U pairs within the lncRNA and miRNA complementary regions were considered miRNA targets.

### Analysis of the positional relationship between lncRNAs and mRNAs

lncRNAs regulate target genes via proximal (*cis*) or remote (*trans*) control. lncRNAs were identified as *cis* if they were located upstream of the mRNA or within 20 k downstream. Beyond this range, *trans* lncRNAs did not rely on the locational relationship, and the binding energy had to be calculated. RNAplex software was used to analyse the binding energy of the lncRNAs and mRNAs. If the binding energy was < 30, then the lncRNA was identified as *trans* and the mRNA adjacent to the lncRNA was screened as its target gene. Spearman and Pearson correlation coefficients were used to screen target genes, with eligibility as a target defined by a Spearman correlation coefficient ≥ 0.6 and a Pearson correlation coefficient ≥ 0.6.

### lncRNA identification as miRNA precursors

To identify lncRNAs acting as precursors of known or novel miRNAs, lncRNAs were aligned with precursors of known miRNAs in the miRBase 21.0 database (http://www.mirbase.org/) with the NCBI Basic Local Alignment Search Tool (BLAST; https://blast.ncbi.nlm.nih.gov/Blast.cgi) using the default parameters. lncRNAs homologous to miRNA precursors with > 90% coverage were defined as miRNA precursors.

### Expression analysis

The expression levels of all transcripts, including lncRNAs and mRNAs, in the NF and EF buds were quantified as the fragments per kilobase of exon per million fragments mapped (FPKM) using the Cuffdiff programme from the Cufflinks package [24, 26–28]. The multiread and fragment bias correction methods embedded in Cufflinks were adopted to improve the accuracy of expression level estimations. DEGs were identified using the DESeq package with an adjusted *P*-value of 0.01 and a fold change of at least 1.2 [28].

### Analysis of lncRNAs near coding genes and prediction of lncRNA families

To better annotate and understand the functions of the predicted lncRNAs, the lncRNAs were classified into different families according to their evolutionary ancestor using the Rfam database (http://rfam.xfam.org/) and INFERNAL software (http://eddylab.org/infernal/) with the default parameters [24, 25, 29].

### Function prediction and lncRNA and mRNA enrichment

All assembled transcripts were annotated using the publicly available Gene Ontology (GO; http://www.geneontology.org) protein database. Gene function enrichment was calculated based on a hypergeometric distribution. The phyper function in R was used to analyse the *P*-value for each function theme:

$$ \mathrm{P}=1\hbox{-} \sum \limits_{i=0}^{m-1}\frac{\left(\begin{array}{c}M\\ {}i\end{array}\right)\left(\begin{array}{c}N-M\\ {}n-i\end{array}\right)}{\left(\begin{array}{c}N\\ {}n\end{array}\right)} $$

Smaller *P*-values were associated with greater enrichment of the candidate genes in a given function theme (https://en.wikipedia.org/wiki/Hypergeometric_distribution).

### Orthologue analysis of genes involved in floral transition in *C. bungei*

*Arabidopsis* genes related to floral transition were obtained from previous studies [30–36]. The gene sequences were downloaded from The Arabidopsis Information Resource (http://www.arabidopsis.org/). The putative orthologue pairs from *Arabidopsis* were analysed using BLAST [28]. Based on the data obtained from BLAST, we identified the homologous genes of interest in *C. bungei* (Additional file 1).

### Quantitative real-time (qRT)-PCR and correlation analysis of expression trends

Total RNA was extracted using an RNA reagent kit (RN38; Aidlab Biotechnology, Beijing, China) according to the manufacturer’s protocol and were treated with RNase-free DNase I (Takara, Dalian, China) to remove genomic DNA contamination. The qRT-PCR analyses were conducted with a 7500 Real-Time PCR System (Applied Biosystems, CA, USA) using the SYBR Premix Ex Taq Kit (TaKaRa, Dalian, China) following the manufacturer’s instructions. Relative expression levels were calculated using the 2^–ΔΔCt^ method. *Cbu-actin* and U6 were amplified as an endogenous control [18]. All the primers are shown in Additional file 2. We test the correlation of expression (CEG) between lncRNAs/miRNAs and lncRNAs/mRNAs by using the Pearson correlation coefficient. The Pearson correlation coefficient was calculated by COR() in R [37].

### Data access

The stranded RNA-seq datasets are available in the Sequence Read Archive database in NCBI (accession number: SRP120718).

## Results

### Morphological analysis of EF and NF buds from *C. bungei*

The major distinguishing characteristic of the EF variety is its one-year juvenile period; in contrast, the NF variety has a minimum juvenile period of five years. To study the external and internal morphologies of EF and NF buds in different growth phases, we collected buds from two-year-old EF and NF plants. Changes in bud appearance were recorded and paraffin sections were produced to observe the internal morphologies of the buds in the corresponding stages.

**Fig. 1:**
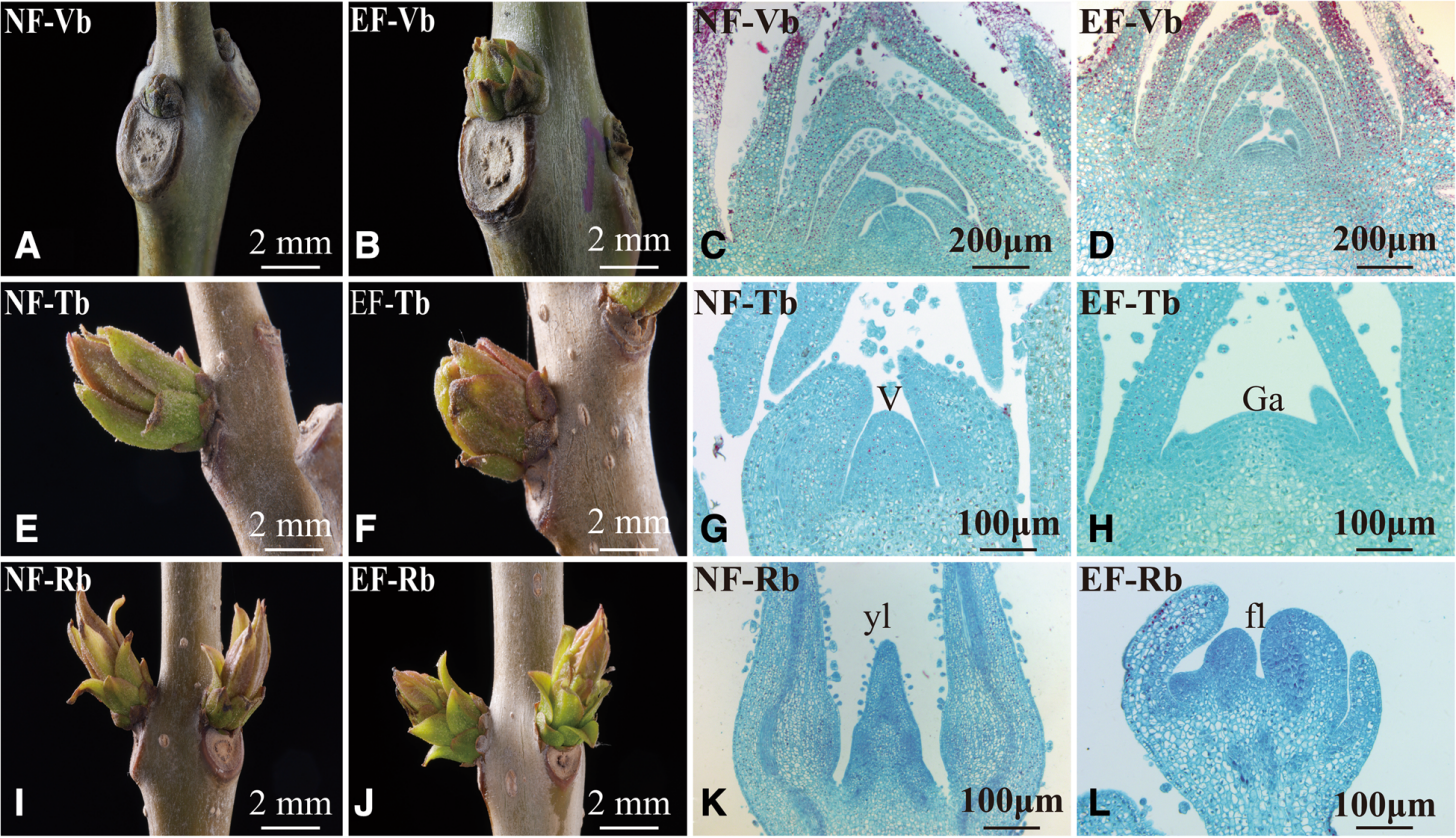
External and internal morphologies of buds from early flowering (EF) and normal flowering (NF) *C. bungei*. **a**–**d** Images and sections of vegetative buds (Vbs) from NF and EF varieties. **a** Image of a Vb from the NF variety (NF-Vb). **b** Image of a Vb from the EF variety (EF-Vb). **c** Section of an NF-Vb. **d** Section of an EF-Vb. **e**–**h** Images and sections of transition buds (Tbs) from the NF and EF varieties. **e** Image of an NF-Tb. **f** Image of an EF-Tb. **g** Section of an NF-Tb, note the bulged vegetative apex (V). **h** Section of an EF-Tb, note the flat generative apex (Ga). **i**–**l** Images and sections of reproductive buds (Rbs) from the NF and EF varieties. **i** Image of an NF-Rb. **j** Image of an EF-Rb. **k** Section of an NF-Rb, note the leaf primordium (lp). **l** Section of an EF-Tb, note the flower primordium (fl)

In the early stage, the buds from the EF and NF plants were vegetative buds (Vbs). Although the EF-Vbs were generally larger than the NF-Vbs (Fig. 1a, b), no differences were observed in the sections (Fig. 1c, d). With continued plant growth, the NF buds generally became longer and thinner than the swollen EF buds (Fig. 1e, f). Conversely, the internal morphologies of the buds in this stage differed substantially. The apex in the NF buds bulged, indicating that they were still in the vegetative growth stage (Fig. 1g). In comparison, a flattened generative apex, which marked the floral transition, developed in the EF buds (Fig. 1h), which were considered transition buds (Tbs).

The morphological differences between the EF and NF buds became more evident over time. The NF buds remained in the vegetative stage, with elongated leaf primordia (Fig. 1i, k), whereas the EF buds developed into reproductive buds (Rbs) that had completed the transformation from vegetative growth to reproductive growth and had begun differentiation into the flower primordia (fl) (Fig. 1j, l). Notably, even though floral transition was never observed in the NF buds, the NF buds collected in the period corresponding to floral transition are henceforth called Tbs or Rbs for convenience.

Figure 1 shows the external and internal morphologies of the EF and NF buds in the three growth phases. The appearance of the generative apex marked the occurrence of floral transition, whereas the initiation of the flower primordia marked the completion of floral transition. The results indicated that the early vegetative-reproductive transition only occurred in the EF buds, not the NF buds. The EF and NF samples were divided into six groups according to these results for the following experiments.

### High-throughput sequencing

**Fig. 2:**
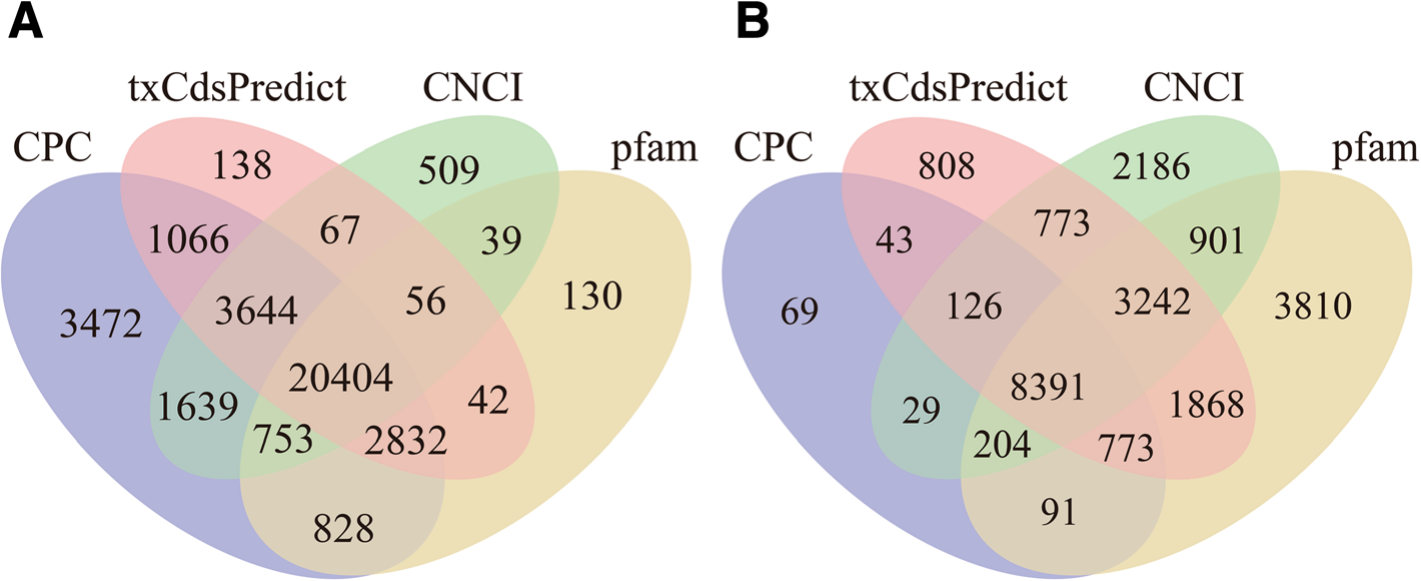
Venn plots showing mRNA and lncRNA predictions. **a** Predicted coding genes. **b** Predicted non-coding genes. CPC1, txCdspredict, CNCI2, and Pfam3 were used to predict the mRNAs and lncRNAs. The parameters of the three programmes used for prediction are as follows: CPC threshold, ≥ 0 = mRNA, < 0 = lncRNA; txCdspredict threshold, ≥ 500 = mRNA, < 500 = lncRNA; CNCI threshold, ≥ 0 = mRNA, < 0 = lncRNA. Pfam is a protein-coding sequence database. Transcripts were confirmed as lncRNAs or mRNAs through prediction by at least three of the four methods

We performed 150-bp paired-end sequencing on raw reads using the Illumina HiSeq 4000 platform by BGI. The total initial reads were processed with in-house Perl scripts. The reads with more than 0.02% unknown bases, the reads with more than 0.68% adapter bases and the reads with more than 0.17% low-quality bases were removed, yielding 13G high-quality (Phred-like Q20, Q30, and GC content) raw reads [20, 38, 39] (Additional file 3). The reads with more than 0.02% rRNA and the reads with more than 28.74% duplications were removed from the 13G raw reads, yielding 9G clean reads (Table 1). All the following analyses were based on the 13G raw reads and 9G clean reads (Additional file 4). From our data, more than 70% of the lncRNAs and mRNAs were mapped to the *C. bungei* genome [40, 41]. In total, 12,532 lncRNAs and 26,936 mRNAs were obtained (Fig. 2), with 82.4% of the lncRNA transcripts distributed within the length range of 0–1000 nt, and 74.1% of the mRNA transcripts were shorter than 2500 nt. Most of the lncRNAs (81.4%) contained one to two exons, whereas most of the mRNAs (59.2%) contained three to more than ten (11.2%) exons (Additional file 5) [9].

**Table 1:** Statistical data of the RNA-SEQ

	NF			EF		
	Vb	Tb	Rb	Vb	Tb	Rb
Raw reads	1,317,350,57.3	131,393,398	1.313.956.05.3	131,925,484	1.317.243.187	1,315,289,37.3
Clean read	88,179,437.33	88,524,307	89,997,175.30	90,551,314	83,876,034.60	87,239,646
Total Mapping Ratio(lncRNAs)	75.91%	76.00%	76.25%	78.19%	75.51%	76.66%
Total Mapping Ratio (mRNAs)	73.93%	74.24%	74.98%	74.51%	73.29%	75.52%
Uniquely Mapping Ratio (lncRNAs)	71.90%	71.99%	72.26%	74.23%	71.66%	72.55%
Uniquely Mapping Ratio (mRNAs)	26.07%	25.76%	25.02%	25.49%	26.71%	24.48%

### Prediction of lncRNA transcripts as miRNA precursors

**Fig. 3:**
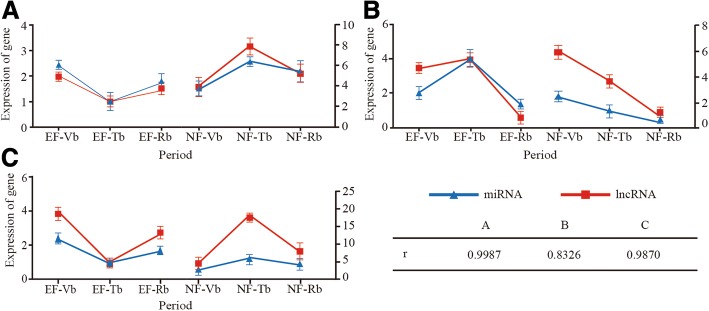
The correlation coefficients of expression of lncRNAs and miRNAs between the EF and NF varieties during three developmental periods. **a** The expression of LXLOC.019956-miR156 between the EF and NF varieties during the three developmental periods. **b** The expression of LXLOC.024876-miR160 between the EF and NF varieties during the three developmental periods. **c** The expression of LXLOC.008074-miR171 between the EF and NF varieties during the three developmental periods. The red line with a square represents the expression of lncRNA, and the blue line with a triangle represents the expression of miRNA. r is the correlation coefficient of expression of the three “co-expression” pairs

lncRNAs that matched miRNA precursors with more than 90% similarity were selected as potential precursors of the corresponding miRNAs, and seven lncRNAs were identified. Of these, three were known lncRNAs and four were unknown lncRNAs, including precursors for six miRNA families (miR414, miR466, miR171, miR156, miR2916, and miR160) (Table 2, Additional file 6). To further study the correlations between lncRNAs and miRNAs, we analyzed their CEGs in the EF and NF samples. In-depth research was performed on three of the six miRNA families: miR156, miR160, and miR171. The results showed that lncRNAs and miRNAs have similar positive correlations with *r* > 0.8 (Fig. 3), implying that lncRNAs may be able to indirectly regulate the floral transition as miRNA precursors.

**Table 2:** List of the lncRNAs transcripts predicted to be miRNA precursors

LncRNA ID	miRNA ID	LncRNA length/bp
lcl|Mguttatus_Migut.J01166.1_dup1	ath-MIR414	323
lcl|Mguttatus_Migut.I00370.1_dup1	gga-MIR466	788
lcl|Mguttatus_Migut.M01324.1_dup1	vvi-MIR171d	1166
lcl|Mguttatus_Migut.M01324.1_dup1	mes-MIR171c	1166
lcl|Mguttatus_Migut.M01324.1_dup1	vvi-MIR171j	1166
LXLOC_019956	aqc-MIR156b	591
LXLOC_023659	peu-MIR2916	1461
LXLOC_024876	ssl-MIR171a	1489
LXLOC_019956	mes-MIR156h	591
LXLOC_019956	mes-MIR156i	591
LXLOC_019956	mes-MIR156j	591
LXLOC_008074	mes-MIR160d	4648
LXLOC_008074	mes-MIR160h	4648
LXLOC_008074	cpa-MIR160c	4648
LXLOC_008074	cpa-MIR160f	4648

### lncRNA target prediction

**Fig. 4:**
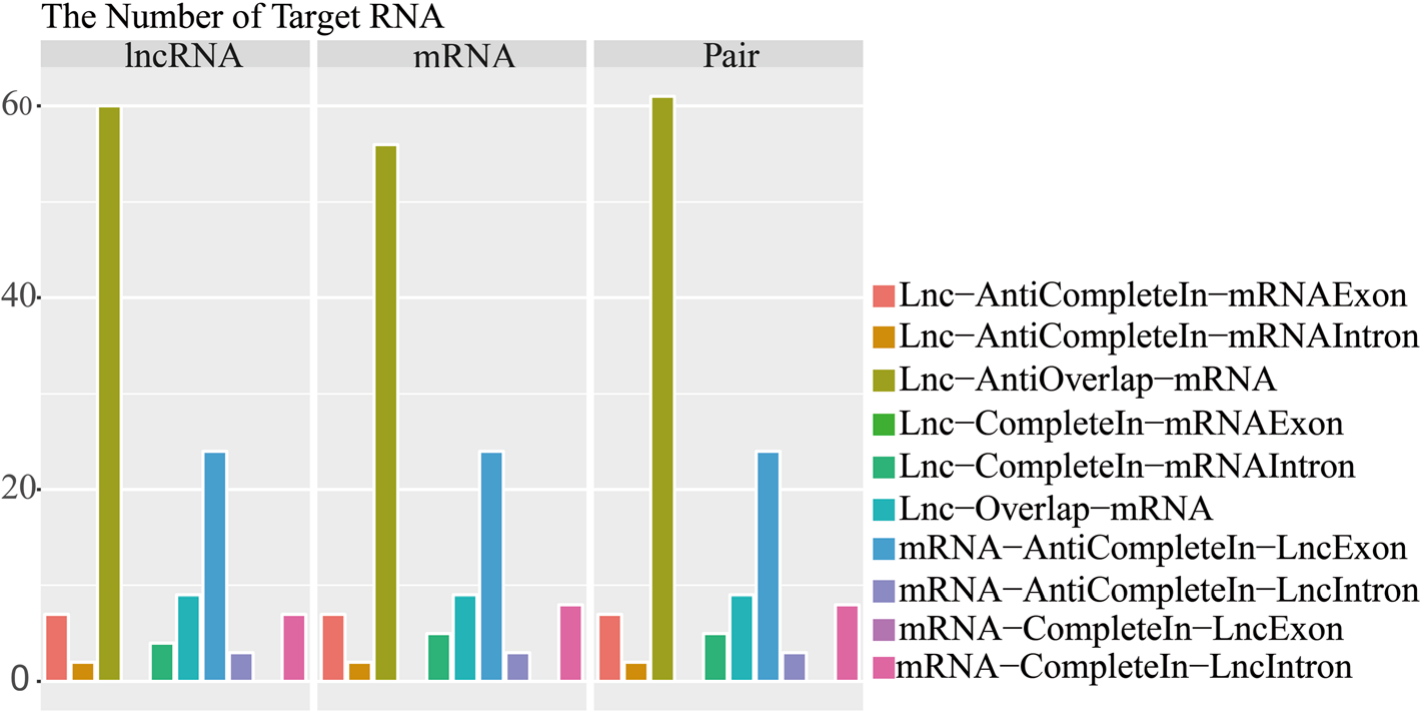
Column plot showing the number of lncRNA and mRNA interaction pairs in different types. Lnc-AntiCompleteIn-mRNAExon: the lncRNA and mRNA are in different chains and the lncRNA is completely within the exon of the mRNA; Lnc-AntiCompleteIn-mRNAIntron: the lncRNA and mRNA are in different chains, and the lncRNA is completely within the intron of the mRNA; Lnc-Antioverlap-mRNA: the lncRNA and mRNA are in different chains but partially overlap; Lnc-CompleteIn-mRNAExon: the lncRNA is in the same chain as the mRNA, and the lncRNA is completely within the exon of the mRNA; Lnc-CompleteIn-mRNAIntron: the lncRNA is in the same chain as the mRNA, and the lncRNA is completely within the intron of the mRNA; Lnc-Overlap-mRNA: the lncRNA is in the same chain as the mRNA, and the lncRNA and mRNA partially overlap; mRNA-AntiCompleteIn-LncExon: the lncRNA and mRNA are in different chains, and the mRNA is completely within the exon of the lncRNA; mRNA-AntiCompleteIn-LncIntron: the lncRNA and mRNA are in different chains, and the mRNA is completely within the intron of the lncRNA; mRNA-CompleteIn-LncExon: the lncRNA is in the same chain as the mRNA, and the mRNA is completely within the exon of the lncRNA; mRNA-CompleteIn-LncIntron: the lncRNA is in the same chain as the mRNA, and the mRNA is completely within the intron of the lncRNA

In total, 119 lncRNA-mRNA pairs were identified using this method, including 61 Lnc-Antioverlap-mRNA pairs. Interestingly, these 61 pairs included 60 lncRNAs and 56 mRNAs, indicating that pairing did not occur exclusively in a one-to-one manner. No Lnc-CompleteIn-mRNA exon pairs were found (Fig. 4; Additional file 7).

### lncRNA-mRNA pairs involved in floral transition

**Fig. 5:**
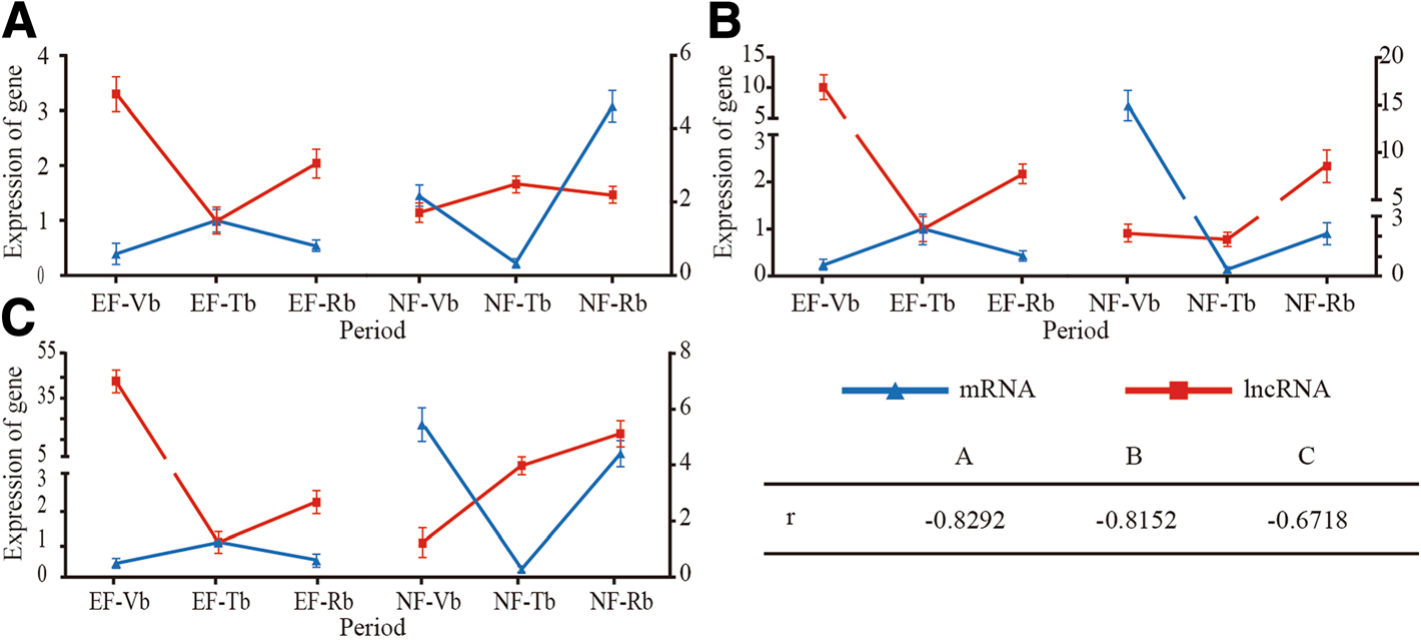
The correlation coefficients of expression of lncRNAs and mRNAs between the EF and NF varieties during three developmental periods. **a** The expression of LXLOC.019079-Cbu.gene.1092 between the EF and NF varieites during the three developmental periods. **b** The expression of LXLOC.017817-Cbu.gene.24144 between the EF and NF varieties during the three developmental periods. **c** The expression of LXLOC.030659-Cbu.gene.7250 between the EF and NF varieties during the three developmental periods. The red line with a square represents the expression of lncRNA, and the blue line with a triangle represents the expression of mRNA. r is the correlation coefficient of expression of the three “co-expression” pairs

To better understand the roles of lncRNAs, we analysed the lncRNA-mRNA interactions and predicted the lncRNA protein-coding targets (Additional file 7). Seven of the mRNAs were related to flowering: Cbu.gene.18564, Cbu.gene.2167, Cbu.gene.190, Cbu.gene.24144, Cbu.gene.7250, Cbu.gene.20428, and Cbu.gene.1092 (Table 3). To further study the correlations of the lncRNA-mRNA pairs, we analysed the correlation coefficients of lncRNAs and mRNAs between the EF and NF samples during three developmental periods. In-depth research was performed on three lncRNAs-mRNAs pairs: LXLCO_019079/Cbu.gene.1092, LXLOC_017817/Cbu.gene.24144 and LXLOC_030659/Cbu.gene.7250. The expression trends of the mRNAs were highest in EF-Tbs. These results were consistent with the expression patterns of the homologous genes in *Arabidopsis* [31, 33]. Based on the overall trend, the three pairs exhibited negative correlations with *r* < − 0.6. While the observed expression correlations between lncRNAs and their co-expressed genes are highly intriguing, whether they reflect true regulatory relationships requires further testing (Fig. 5).

**Table 3:** The lncRNAs-mRNA pairs involving in floral transition

LncRNA	mRNA	lncRNA length/bp	mRNA length/bp	Annotation
LXLOC_006261	Cbu.gene.20428	2519	975	NAC homologous gene
LXLOC_019079	Cbu.gene.1092	1598	1657	TRY homologous gene
LXLOC_019953	Cbu.gene.190	943	1872	NF-YC homologous gene
LXLOC_017817	Cbu.gene.24144	1392	641	SOC1 homologous gene
LXLOC_030659	Cbu.gene.7250	766	922	SOC1 homologous gene
LXLOC_025313	Cbu.gene.18564	694	1300	AP2 homologous gene
LXLOC_004449	Cbu.gene.27201	1877	738	AP2 homologous gene

### GO analysis of DEGs and target genes of DELs

**Fig. 6:**
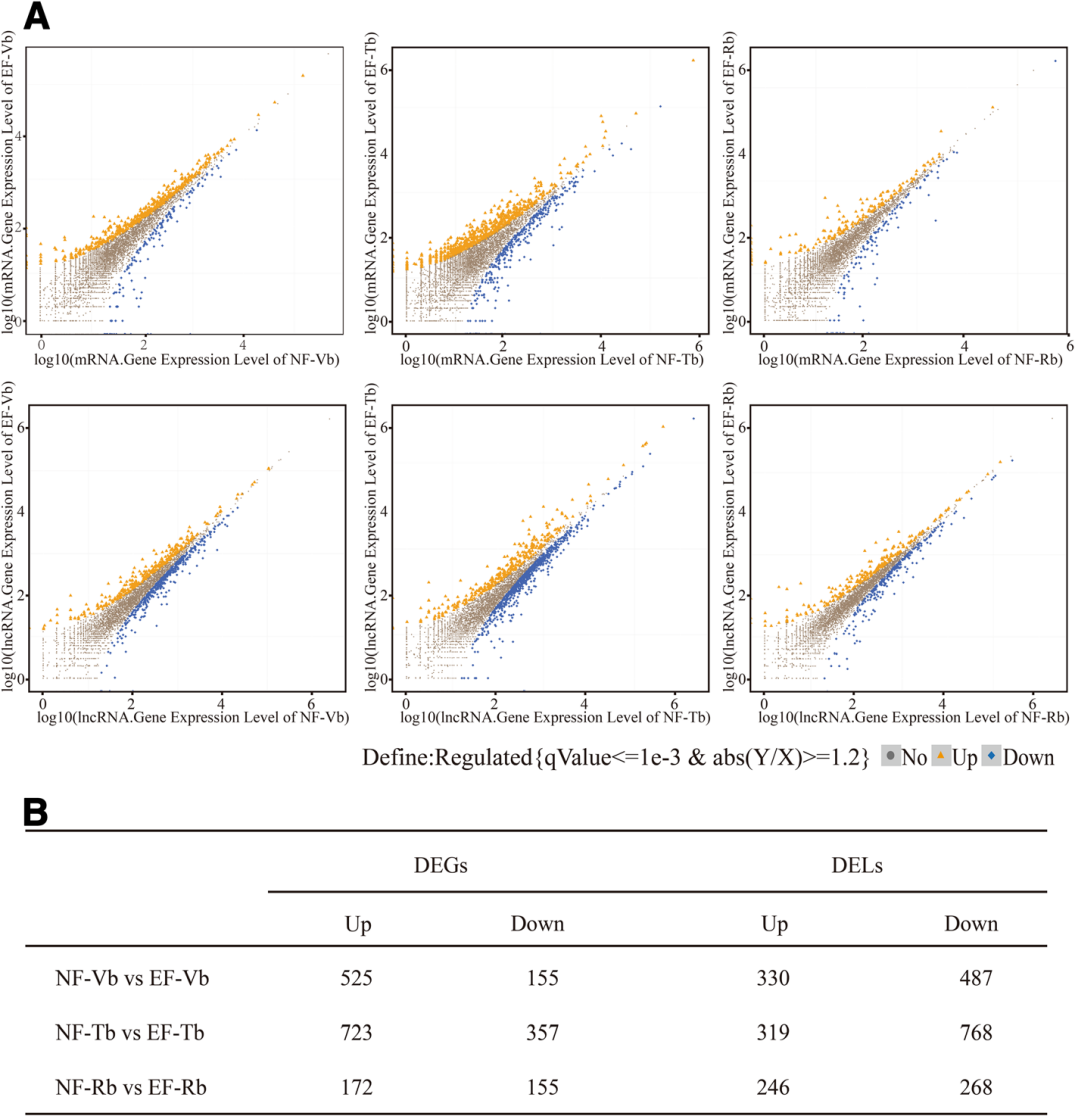
DEGs and DELs in NF and EF *C. bungei*. **a** Scatter plot of the comparative results of the log-transformed gene expression levels (Log10 FPKM) and DEG or DEL distributions between the NF and EF samples. The orange rectangles represent up-regulated genes, and the blue diamonds represent down-regulated genes. **b** Summary of the numbers of up- or down-regulated mRNAs and lncRNAs in NF-Vb vs. EF-Vb, NF-Tb vs. EF-Tb, and NF-Rb vs. EF-Rb

In total, 680 DEGs and 817 DELs were identified in NF-Vbs versus EF-Vbs, 1089 DEGs and 1087 DELs were identified in NF-Tbs versus EF-Tbs, and 306 DEGs and 514 DELs were identified in NF-Rbs versus EF-Rbs (Fig. 6, Additional file 8). Fig. 6b shows the numbers of up- or down-regulated mRNAs and lncRNAs.

Eighteen DEGs related to flower development were identified in all three groups (Table 4). However, genes regulating the vegetative-reproductive transition were identified only in NF-Vbs versus EF-Vbs. These genes included one *SPL* gene (Cbu.gene.13552) and two novel genes (Cbu.gene.5215 and Cbu.gene.12342). Cbu.gene.13552, which exhibited greater expression in EF-Vbs than in NF-Vbs, was predicted to be a target of miR529 (psRNATarget, Additional file 9), which is consistent with previous reports of *SPL* [42]. These results suggested that, although the morphological shift of the floral transition could only be observed in EF-Tbs, the floral transition had started in EF-Vbs at a molecular level. Overall, the Vb phase is a critical stage for floral transition gene regulation.

**Table 4:** Parital Gene ontology associated with flower development

Gene ID	Term	GO ID
Cbu.gene.11993	floral whorl development	GO:0048438
	floral organ development	GO:0048437
	flower development	GO:0009908
Cbu.gene.13552	regulation of flower development	GO:0009909
Cbu.gene.16262	pollen development	GO:0009555
	pollen exine formation	GO:0010584
	pollen wall assembly	GO:0010208
Cbu.gene.16343	flower development	GO:0009908
Cbu.gene.17110	pollen development	GO:0009555
Cbu.gene.17779	recognition of pollen	GO:0048544
	pollen-pistil interaction	GO:0009875
	flower development	GO:0009908
Cbu.gene.18350	floral organ development	GO:0048437
Cbu.gene.20397	pollen development	GO:0009555
	pollen exine formation	GO:0010584
	pollen wall assembly	GO:0010208
Cbu.gene.20428	flower development	GO:0009908
Cbu.gene.21602	flower development	GO:0009908
	floral whorl development	GO:0048438
	floral organ development	GO:0048437
Cbu.gene.24436	recognition of pollen	GO:0048544
	pollen-pistil interaction	GO:0009875
Cbu.gene.24775	floral whorl development	GO:0048438
	floral organ development	GO:0048437
	stamen development	GO:0048443
	androecium development	GO:0048466
Cbu.gene.26021	flower development	GO:0009908
Cbu.gene.5215	regulation of flower development	GO:0009909
Cbu.gene.6097	pollen exine formation	GO:0010584
	pollen wall assembly	GO:0010208
	pollen development	GO:0009555
Cbu.gene.6356	shoot system development	GO:0048367
Cbu.gene.8663	flower development	GO:0009908
Cbu.gene.26687	floral whorl development	GO:0048438
	floral organ development	GO:0048437
	stamen development	GO:0048443
	androecium development	GO:0048466

### DEGs involved in floral transition

**Fig. 7:**
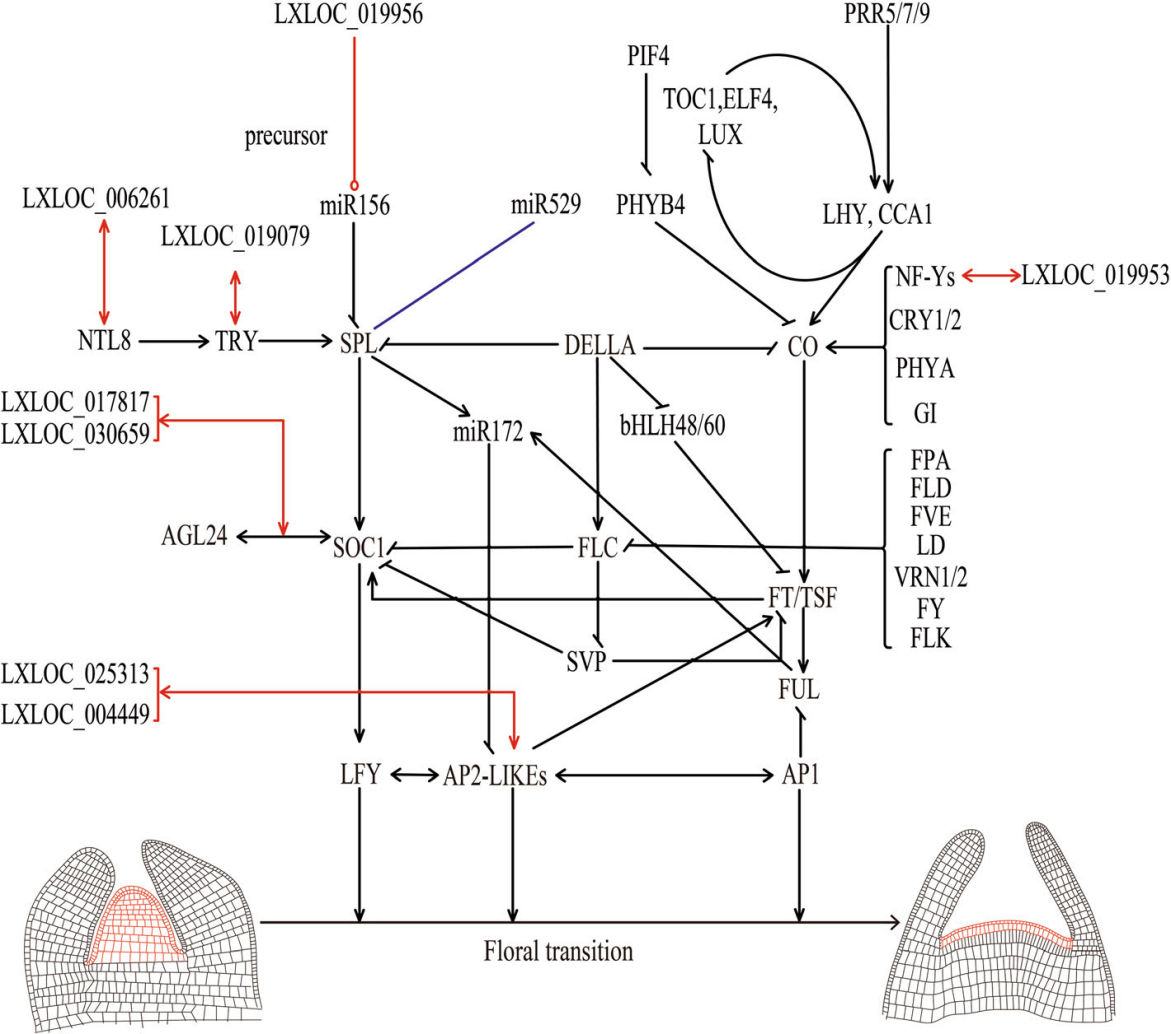
Floral transition network in *Arabidopsis*. The red text represents the main genes in the floral transition network of *Arabidopsis*. The black lines represent the relationships in *Arabidopsis*. The red lines represent the relationships implied by this study in *C. bungei*. The singe-headed arrows indicate that gene expression is promoted. The double-headed arrows indicate the lncRNA-mRNA pairs in *C. bungei*. The curly brackets with arrows indicate that the expression of the genes in the brackets is promoted. The short lines indicate negative gene expression. The red lines with rings indicate gene precursors. The bottom figure shows the floral transition. In this bottom figure, the red areas represent the shoot apical meristem, the left image shows the vegetative growth terminus, and the right image shows the reproductive growth terminus

**Fig. 8:**
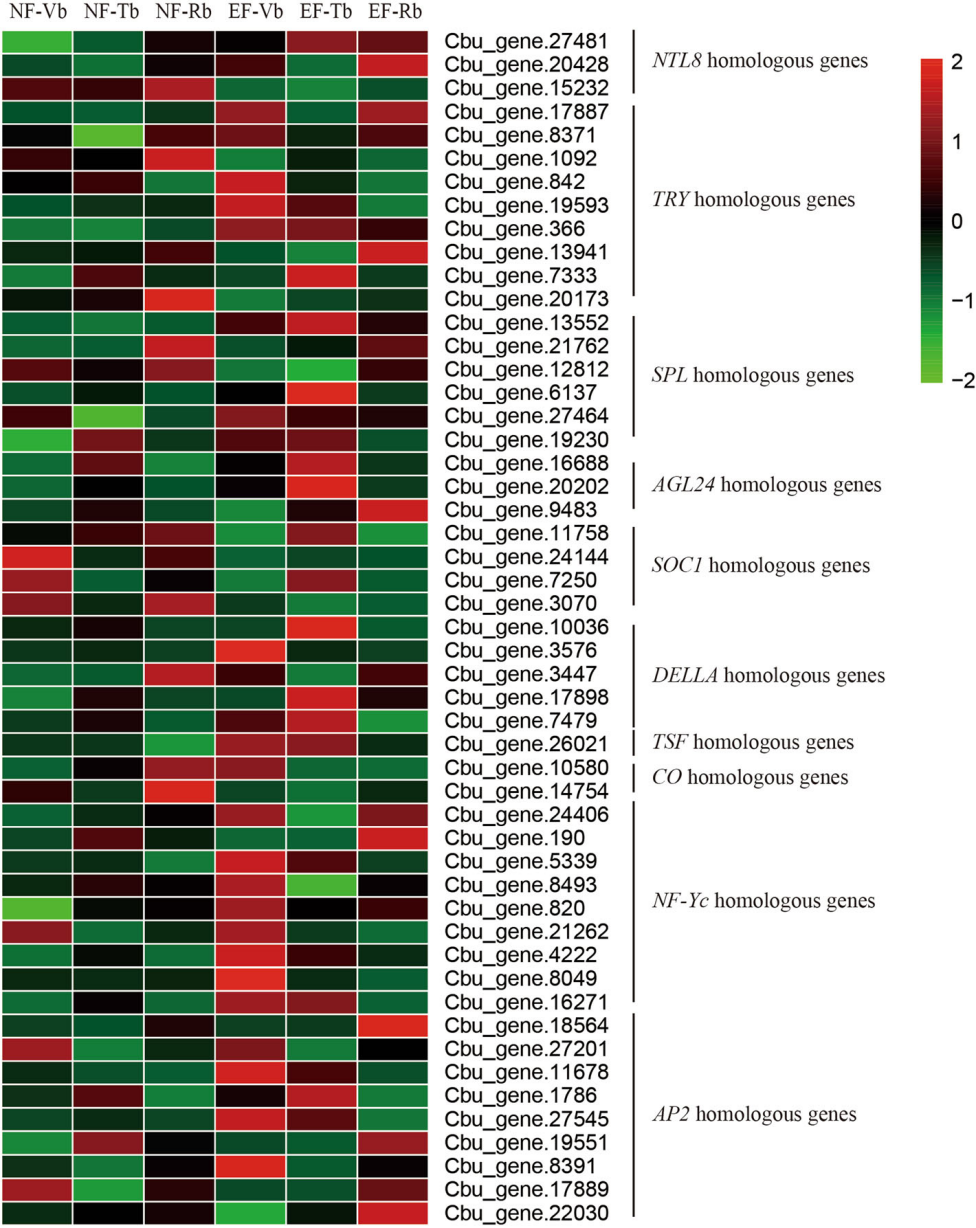
Expression analysis of the floral transition homologous genes in *C. bungei*. Vertical bars indicate homologous genes from *Arabidopsis*. The text alternates between black and red for clarity

Floral transition has been studied extensively in *Arabidopsis thaliana* and more than 100 floral transition-related genes have been reported (Fig. 7, Additional file 1). Many homologous genes related to floral transition were found by the BLAST search against the transcriptome data. In addition to the aforementioned genes, several additional genes related to flowering were differentially expression in EF and NF buds (Fig. 8, Additional file 10). Five *SPL* homologous genes were found in this study, and these genes exhibited greater expression in EF-Vbs than in NF-Vbs. *SPLs* include very large gene families that mainly promote flowering time via age pathways [43, 44]. *SPLs* promote floral transition by activating the expression of several other genes, such as *FT* genes*.*

Cbu.gene.26021 is homologous with genes in the *FT* family. Cbu.gene.26021 exhibited substantially greater expression in EF versus NF buds at all stages. *FT* and *TSF* are *FT* gene family members that work redundantly to promote floral transition in *Arabidopsis* [45]. These results imply that *FT* family-associated flowering transition regulation may exist in *C. bungei*.

The *DELLA* homologous genes, except for Cbu.gene.10036, exhibited greater expression in EF buds than in NF buds. In *Arabidopsis*, *DELLA* is a negative flowering regulation gene that has been found to show lower expression levels in EF buds than in NF buds [46]. Therefore, Cbu.gene.10036 may be the *DELLA* gene in *C. bungei*, or *DELLA* homologs may have a novel role in floral transition in *C. bungei* (Fig. 8, Additional file 10).

### Relationships between DELs and miRNA families

**Fig. 9:**
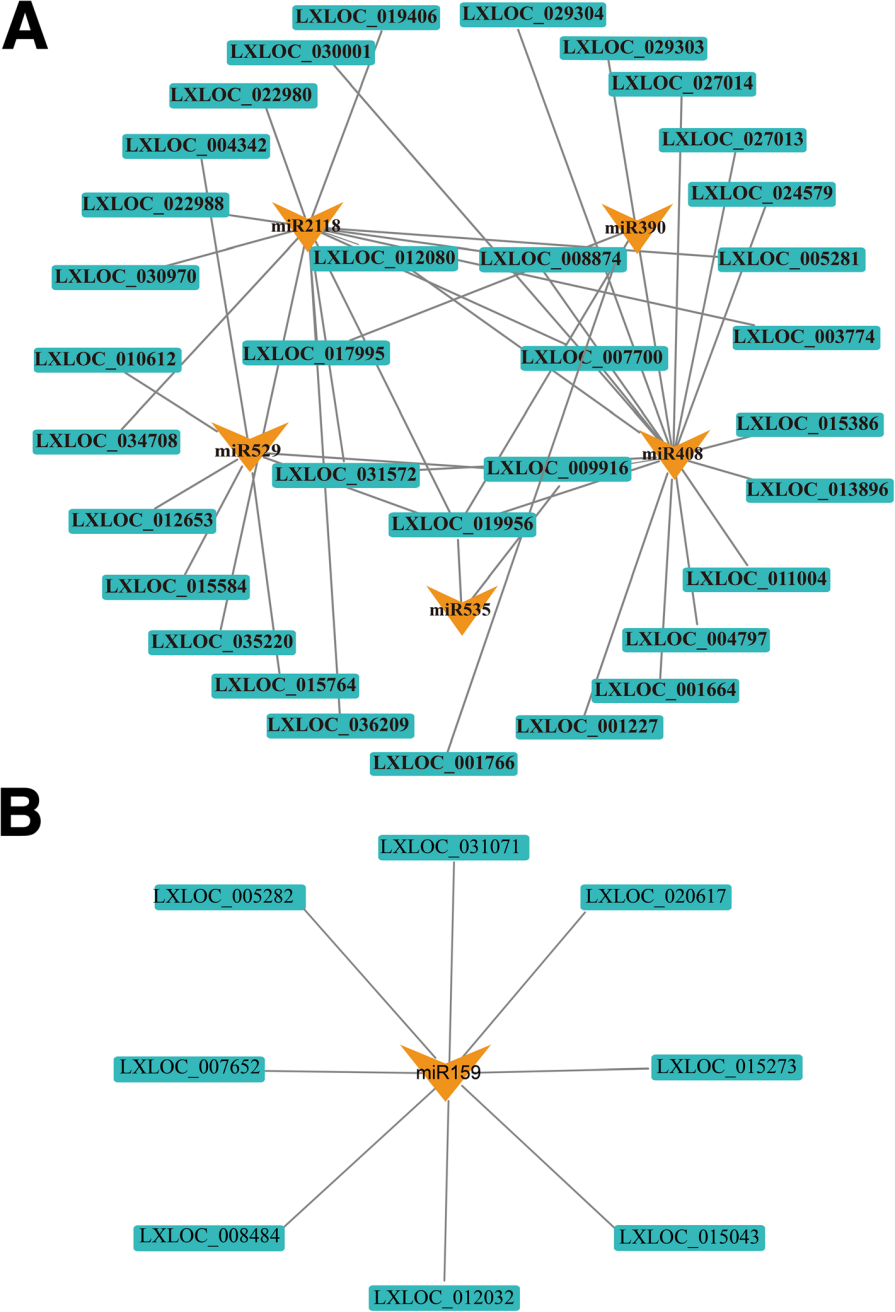
Relationship network of lncRNAs and miRNAs. INFERNAL (http://eddylab.org/infernal/) was used to perform a BLAST search with the lncRNAs of the Rfam database (http://rfam.xfam.org/). In total, 466 lncRNAs may act as decoys of the corresponding miRNAs. The orange triangles indicate the miRNA families. The blue rectangles represent the related lncRNAs. The relationships between the lncRNAs and miRNAs are diverse. **a** Several lncRNAs have relationships with more than one miRNA family. The rings, from outside inward, represent lncRNAs that do not overlap, miRNA families, and lncRNAs that overlap. **b** Nine lncRNAs show a relationship with miR159

The DELs were distributed in 437 families, including 34 known miRNA families (Fig. 9, Additional file 11). These 34 families included 466 related DELs, which may act as decoys of the corresponding miRNAs. The DELs LXLOC_012080, LXLOC_017995, LXLOC_031572, LXLOC_019956, LXLOC_009916, LXLOC_007700, and LXLOC_008874 were related to more than one of the miRNA families miR2118, miR529, miR535, miR408, and miR390 (Fig. 9a, inner layers). In particular, LXLOC_019956 was related to all five miRNA families. These miRNA families are associated with resistance, floral transition, and sucrose metabolism. Most of the lncRNAs were related to unique miRNA families (Fig. 9a, outer layer, B). Together, the DELs and miRNA families formed a complex network to regulate plant development and growth. The relationships between the DELs and miRNA families imply the involvement of plant resistance and sucrose metabolism in floral transition.

### Expression trends of the selected DEGs and DELs in EF and NF buds

**Fig. 10:**
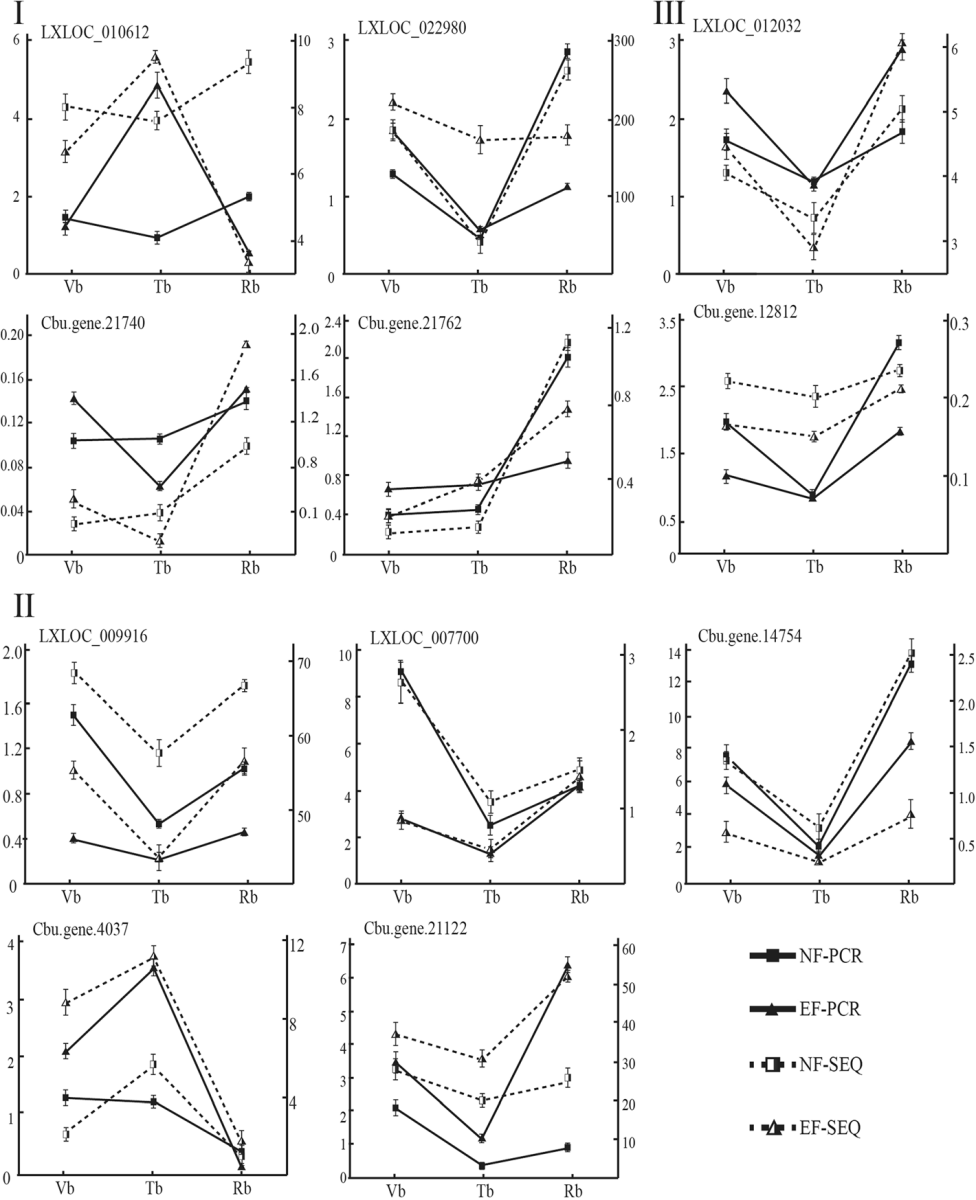
lncRNA and mRNA results from RNA-seq verified by qRT-PCR. **i** lncRNA or mRNA expression levels showing different trends in NF and EF buds; **ii** lncRNA or mRNA expression levels showing the same trend in NF and EF buds; **iii** lncRNA or mRNA expression showing no obvious different trend. The Y-axis represents the relative expression of genes, and the X-axis represents the development periods.  represents the PCR results in NF buds,  represents the PCR results in EF buds,  represents the RNA-seq results in NF buds, and  represents the RNA-seq results in EF buds

We performed RNA-seq and qRT-PCR analysis on five lncRNAs and six mRNAs in NF and EF buds from three growth periods (Fig. 10). The five DELs were related to the five flowering-related miRNA families: miR408, miR398, miR529, miR535, and miR159. The six DEGs were *SPL*, *bHLH*, *LFY*, *CONTANS*, and *STP* (sugar transport protein). The expression trends can be summarized into three categories. I) The expression levels of some lncRNAs or mRNAs, such as LXLOC_010612, LXLOC_022980, Cbu.gene.21740, and Cbu.gene.21762, showed different trends in the NF and EF buds. In the NF buds, LXLOC_010612, which is related to miR529, was highly expressed in Vbs, but LXLOC_010612 expression was decreased in Tbs, and higher levels of LXLOC_010612 were restored in Rbs. This gene showed the opposite expression pattern in EF buds. In EF buds, LXLOC_010612 showed the highest expression level in Tbs and lower expression levels in Vbs and Rbs. Cbu.gene.21762 was a presumed *SPL* family gene. No obvious expression changes were noted in NF/EF-Vbs and NF/EF-Tbs; however, in NF-Rbs, the expression level of Cbu.gene.21762 increased sharply. This increase was minimal in EF buds. Cbu.gene.21740 is a *bHLH* family gene reportedly involved in the jasmonate signalling pathway of plants [47]. From the Vb to Tb phases, the expression of Cbu.gene.21740 decreased in EF buds but increased slightly in NF buds. The different gene expression trends between the EF and NF buds suggest that these genes have a role in the early flowering phenomenon of the EF variety. II) The expression levels of some lncRNAs or mRNAs, such as LXLOC_009916, LXLOC_00700, Cbu.gene.4037, Cbu.gene.14754, and Cbu.gene.21122, showed the same trends in NF and EF buds. III) The expression levels of some lncRNAs or mRNAs showed no obvious trends. Overall, the qRT-PCR and RNA-seq results were in good agreement. The expression levels of the lncRNAs and mRNAs showed period-specific patterns. The mRNAs or lncRNAs that were differentially expressed in the NF and EF buds may contribute to early flowering in the EF variety.

## Discussion

*C. bungei* is an ancient ornamental woody plant in China. The flowering time of this species largely contributes to its commercial value. Floral transition represents a major developmental phase change that transforms the identity of the shoot apical meristem from vegetative to inflorescence. However, partly due to the difficulty associated with selecting the EF buds of *C. bungei*, few studies have investigated the flowering transition of this species. With the development of RNA-seq technology, genome-wide mapping has proven to be a powerful tool for studying the flowering transition in *C. bungei.* Here, we present the first comprehensive analysis of the lncRNAs in *C. bungei* to study floral transition regulation in woody plants. The transcriptomic study was performed with NF and EF varieties to systematically identify the lncRNAs and mRNAs associated with floral transition. In total, 12,532 lncRNAs and 26,936 mRNAs were detected, including 680 DELs and 817 DEGs. Seven lncRNAs were predicted to be miRNA precursors. In addition, 119 lncRNA-mRNA interaction pairs were identified based on localization, function prediction, and binding energy analyses. The results suggest that the molecular regulation of floral transition may occur before the associated morphological changes. Our results offer a deeper understanding of the floral transition mechanism in *C. bungei*, and the selected lncRNAs represent potential targets for future studies.

In this study, most mRNAs, such as *SPL*, *AP2*, *LFY*, *CO*, showed expression patterns similar to those in *Arabidopsis* [30, 34, 36, 37, 48, 49]*.* Cbu.gene.13552, an *SPL* homologous gene, generally showed high expression levels in Vbs. In addition, after a short period of decline, the expression levels of this gene increased during the flower formation stage. Cbu.gene.13552 showed expression trends in *C. bungei* similar to those of *SPLs* in *Arabidopsis* (Fig. 8) [35, 43, 49, 50]. However, the expression trends of some genes in *C. bungei* differed from those of corresponding genes in *Arabidopsis*. For example, except for Cbu.gene.10036, *DELLA* homologous genes in *C. bungei* exhibited greater expression in EF buds than in NF buds. In *Arabidopsis*, *DELLA* is a negative flowering regulation gene that has been shown to exhibit lower expression levels in EF versus NF buds [46, 51]. *FT* and *TSF* are important genes related to floral transition in *Arabidopsis* that regulate floral transition in response to day length (Fig. 8, Additional file 10) [52, 53]. Cbu.gene.18536 is a possible homolog of *FT*, but no differences in the expression levels of this gene were detected between EF and NF buds. Meanwhile, Cbu.gene.26021, a possible homolog of *TSF*, showed a markedly higher expression level in EF buds than in NF buds.

Two main reasons account for these differences in gene expression between *Arabidopsis* and *C. bungei*. First, the sequences and functions of genes in herbs and woody plants may differ. Second, because genes from the same family may have different functions, genes may be annotated inaccurately by imperfect genome annotation information.

In this study, seven lncRNAs were identified as precursors of miRNAs, including three known lncRNAs and four novel lncRNAs (Table 2). LXLOC_019956 represents a potentially intriguing case. The lncRNA LXLOC_019956 was a precursor of miR156 (Additional file 6). According to existing reports, miR156 targets the *SPL* family of genes during the flowering transition [35, 43, 49, 54, 55]. Therefore, LXLOC_019956 may indirectly participate in the floral transition process (Fig. 3).

Moreover, LXLOC_019956 is related to miR529/535/408/390/2118, which are crucial for plant growth and resistance (Fig. 9). miR529/535 have a similar function as miR156 [42, 54, 55]. Cbu.gene.13552, a possible *SPL* gene that was significantly up-regulated in EF-Vbs, was predicted to be a target of miR529 (Additional file 9). Regulation of specific *SPLs* by miR156 and miR529 is important in flower architecture development in monocots, particularly in grasses. However, in this study, miR529s were present in the eudicot *C. bungei*.

In this study, we identified 119 lncRNA-mRNA pairs (Additional file 7). Seven mRNAs from these pairs are encoded by homologs of genes related to floral transition, including *SOC1s*, *AP2s*, *NTL8*, *TRY*, and *NF-YC* (Fig. 7). According to reports, lncRNAs with enhancer-like functions have been discovered, and these lncRNAs also serve as NATs to inhibit the expression of the corresponding mRNAs [21, 56]. Based on the concept that lncRNAs and their regulatory targets may exhibit highly positively or negatively correlated expression patterns, we analysed the expression correlations of lncRNAs and their targets. In our study, we showed three examples of lncRNAs and their negatively correlated mRNAs, including LXLOC_019079 and Cbu.gene.1092 (*TRY* homologous gene), LXLOC_017817 and Cbu.gene.24144 (*SOC1* homologous gene) and LXLOC_030659 and Cbu.gene.7250 (*SOC1* homologous gene) (Fig. 5). The three homologous genes promoted the floral transition in *Arabidopsis* [31]. The identification of three lncRNAs with opposite expression patterns relative to those of their targets warrants further investigation into a possible direct regulatory relationship between lncRNAs and their targets. These results suggest that floral transition-repressive lncRNAs may serve as the hubs of a gene regulatory network, the suppression of which may lead to positive vegetative growth and material development. Overall, lncRNAs have complex functions in organisms. More tests should be carried out to verify the functions of lncRNAs and their mechanisms of action.

We examined the expression levels of five lncRNAs and six mRNAs by qRT-PCR and RNA-seq (Fig. 10). The expression trends of LXLOC_010612, a *bHLH* gene (Cbu.gene.21740), and an *SPL* gene (Cbu.gene.21762) differed significantly between NF and EF buds. LXLOC_010612 was related to miR529, which is important in floral transition. *SPL*s participate in many biological and metabolic processes, such as resistance and floral transition. The expression results suggested that LXLOC_010612, the *bHLH* gene, and the *SPL* gene may be involved in early flowing in the EF variety. However, these genes should be further studied to determine whether they are related to floral transition or flower development.

## Conclusions

In this manuscript, we recorded the external and internal morphologies of EF and NF buds in different growth phases. In this study, 12,532 lncRNAs and 26,936 messenger RNAs (mRNAs) were detected. In addition, 680 DEGs and 817 differentially expressed lncRNAs were detected during the initiation of floral transition. Moreover, the lncRNA LXLOC_019956 was a precursor of miR156, which targets the *SPL* family of genes to enable flowering transition. In addition, seven lncRNA-mRNA interaction pairs were predicted to be involved in floral transition. The present study provides new insights into the role of lncRNAs in the molecular mechanisms underlying the flowering transition. These results can be used to explore the functions of lncRNAs and support further genetic studies of *C. bungei*.

## Additional files

Additional file 1: **Table S1.** List of the floral transition genes in *C. bungei. (XLSX 1035 kb)*

Additional file 2: **Table S2.** List of the primers used in qRT-PCR. (XLSX 13 kb)

Additional file 3: **Table S3.** RNA-seq filter data. (XLSX 21 kb)

Additional file 4: **Table S4.** Summary of the read counts. (XLSX 14 kb)

Additional file 5: **Figure S1.** Distributions of lengths and exon numbers in lncRNAs and mRNAs. A) The distribution of lengths in lncRNAs and mRNAs. B) The distribution of exon numbers in lncRNAs and mRNAs. The pink square represents the distribution in lncRNAs and the blue and red squares represent the distribution in mRNAs. (JPG 6734 kb)

Additional file 6: **Table S5.** miRNA precursor prediction. (XLSX 10 kb)

Additional file 7: **Table S6.** Prediction of the localization relationships of lncRNAs and mRNAs. (XLSX 19 kb)

Additional file 8: **Table S7.** Summary of the DEGs and DELs in different groups. (XLSX 11 kb)

Additional file 9: **Figure S2.** Alignment of Cbu.gene.13552 with miR529. (JPG 1754 kb)

Additional file 10: **Figure S3.** Expression analysis of the homologous genes involved in floral transition in *C. bungei*. The vertical bar indicates that the gene is the homologous gene from *Arabidopsis*. Black red alternation is present to prevent disorder. (JPG 5059 kb)

Additional file 11: **Figure S4.** Interaction networks of lncRNAs and miRNAs. The orange triangle indicates the miRNA families The blue rectangle indicates the related lncRNAs. The relationships among the lncRNAs and miRNAs are diverse. A) The lncRNAs and miRNA families have intersecting relationships. B) The relationships between with the lncRNAs and miRNA families are 1 to a model. C) The relationships between certain lncRNAs and miRNAs intersect. (JPG 4588 kb)

## Abbreviations

AGL24: Agamous-like 24
AP1: Apetala 1
AP2: Apetala 2
bHLH: Basic helix-loop-helix
CCA1: Circadian Clock Associated1
CO: Constans
CRY: Cryptochrome
DEG: Differentially expressed gene
DEL: Differentially expressed LncRNA
EF: Early flowering mutant
ELF: Early flowering
FD: Flowering locus D
fl: Flower primordial
FLC: Flowering Locus C
FPKM: Fragments mapped
FRI: Frigida
FT: Flowering locus T
Ga: Generative apex
GI: Gigantea
Ip: Leaf primordium
LFY: Leafy
LHY: Latelongated Hypocoty L
LncRNA: Long non-coding RNA
LUX: Luxarrhythmo
miRNA: MicroRNA
MYB: Myeloblastosis viral oncogene homolog
NAT: Natural antisense transcript
NF: Normal flowering
NF-Ys: Nuclear factor-Ys
NTL: NAC with transmembrane motif 1 like
PHYB: PhytochromeB
PIF: Phytochrome interacting factor
Rb: Reproductive bud
SOC1: Suppressor of overexpression of CO1
SPL: Squamosa-promoter binding protein-like
sRNA: Small RNA
SVP: Short vegetative phase
Tb: Transition bud
TRY: Triptychon
TSF: Twin sister of FT
Vb: Vegetative bud
VRN: Vernalization
