# Supplementary figures and images for: Genome-wide analysis of long non-coding RNAs in Catalpa bungei and their potential function in floral transition using high-throughput sequencing
Source: BMC Genet. 2018 Sep 20;19:86. doi: 10.1186/s12863-018-0671-2 (PMC6149005; doi:10.1186/s12863-018-0671-2)

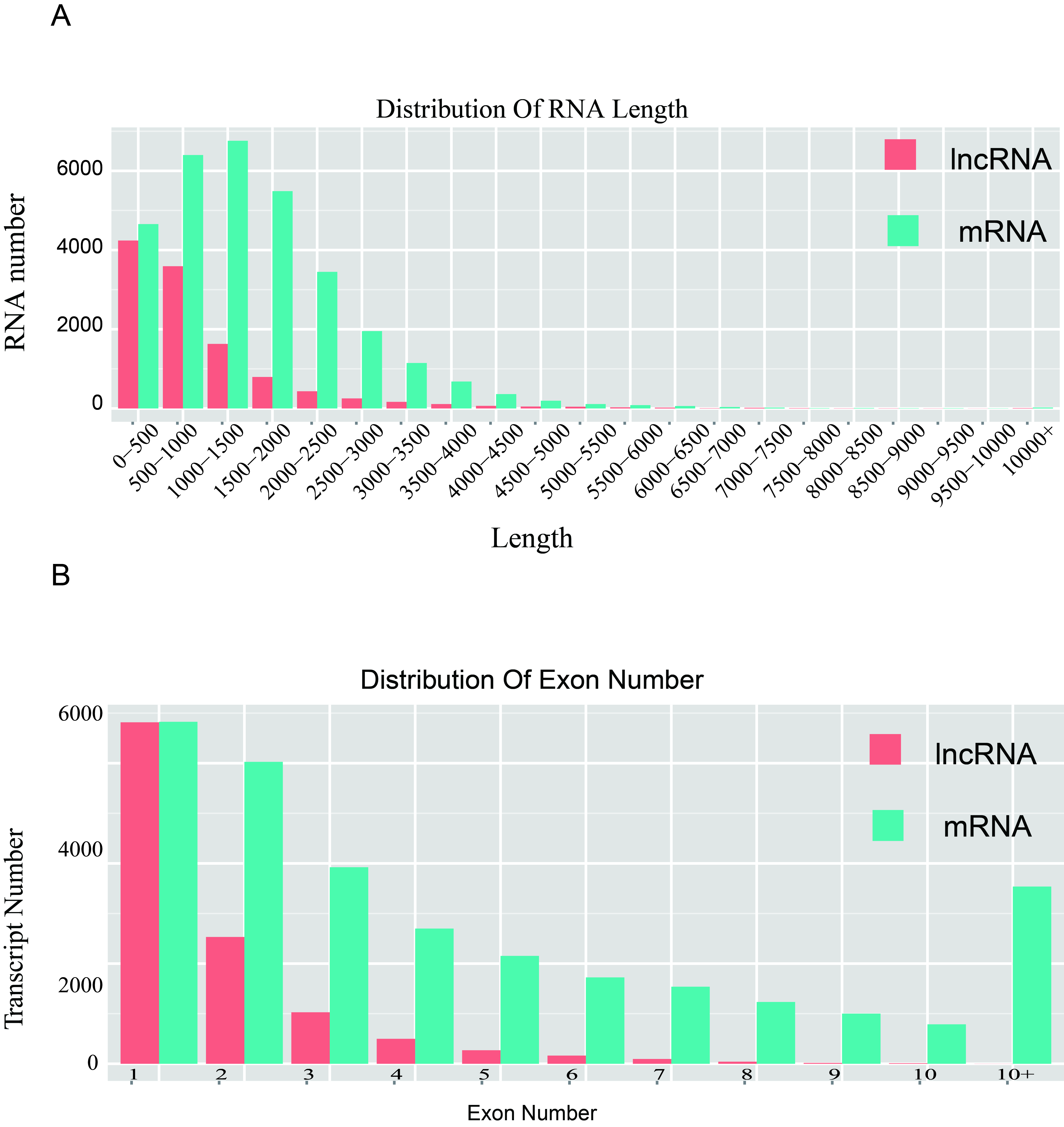

Supplement: Supplementary file 5 — Figure S1. Distributions of lengths and exon numbers in lncRNAs and mRNAs. A) The distribution of lengths in lncRNAs and mRNAs. B) The distribution of exon numbers in lncRNAs and mRNAs. The pink square represents the distribution in lncRNAs and the blue and red squares represent the distribution in mRNAs. (JPG 6734 kb) [file 12863_2018_671_MOESM5_ESM.jpg]

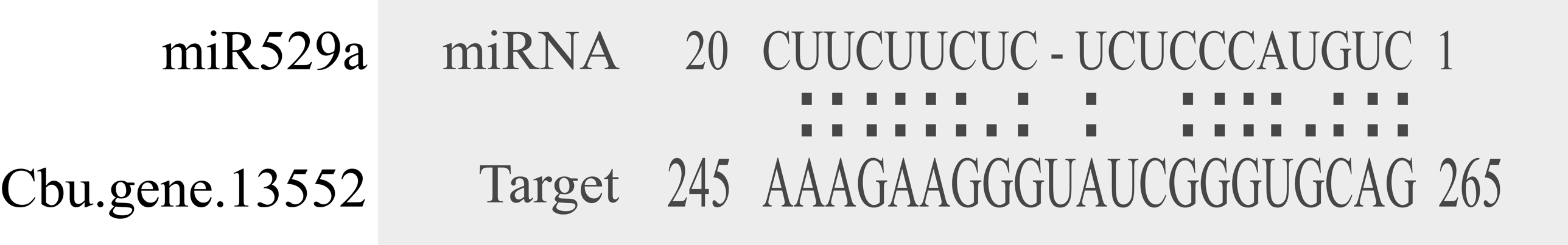

Supplement: Supplementary file 9 — Figure S2. Alignment of Cbu.gene.13552 with miR529. (JPG 1754 kb) [file 12863_2018_671_MOESM9_ESM.jpg]

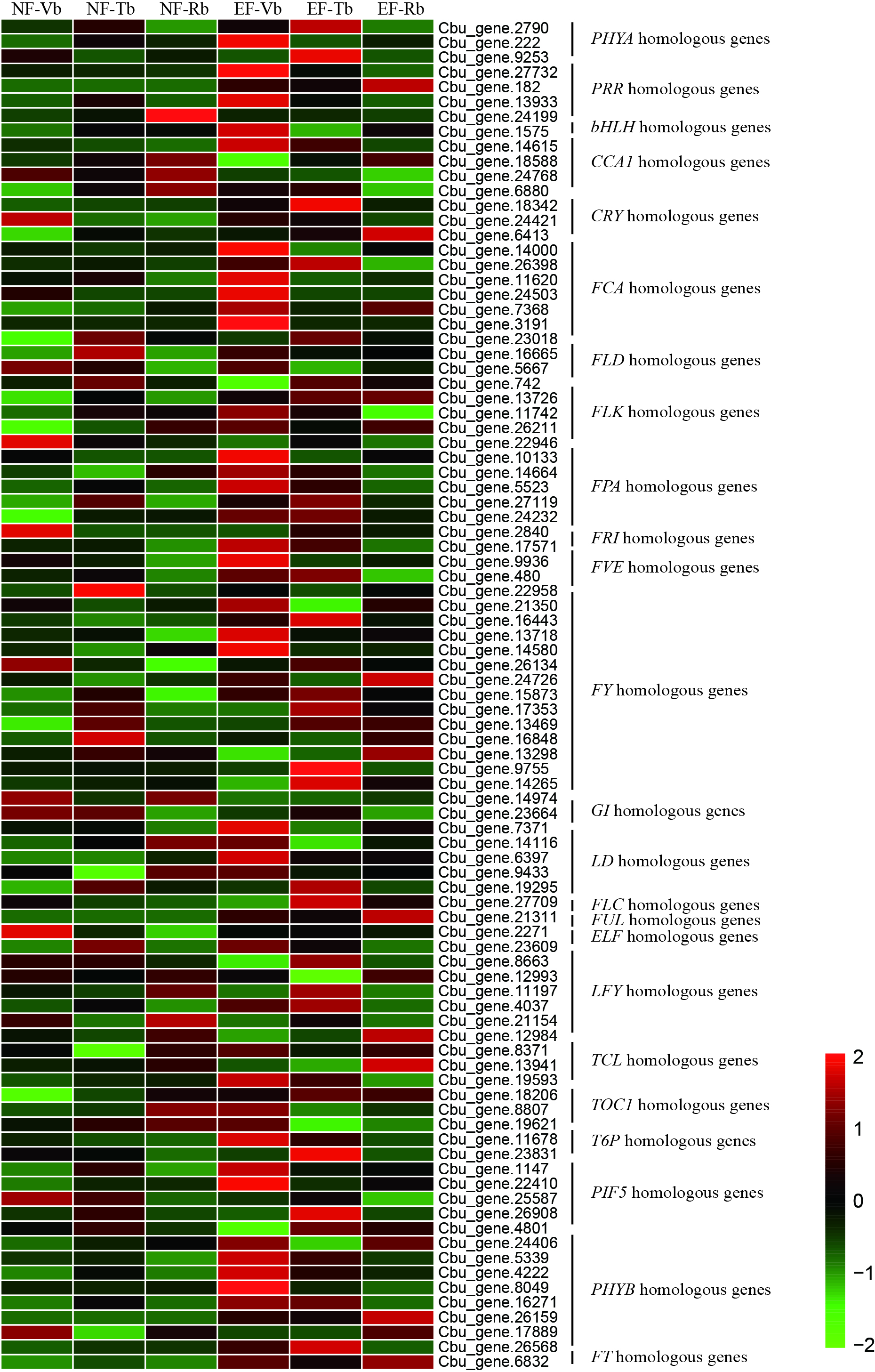

Supplement: Supplementary file 10 — Figure S3. Expression analysis of the homologous genes involved in floral transition in C. bungei. The vertical bar indicates that the gene is the homologous gene from Arabidopsis. Black red alternation is present to prevent disorder. (JPG 5059 kb) [file 12863_2018_671_MOESM10_ESM.jpg]

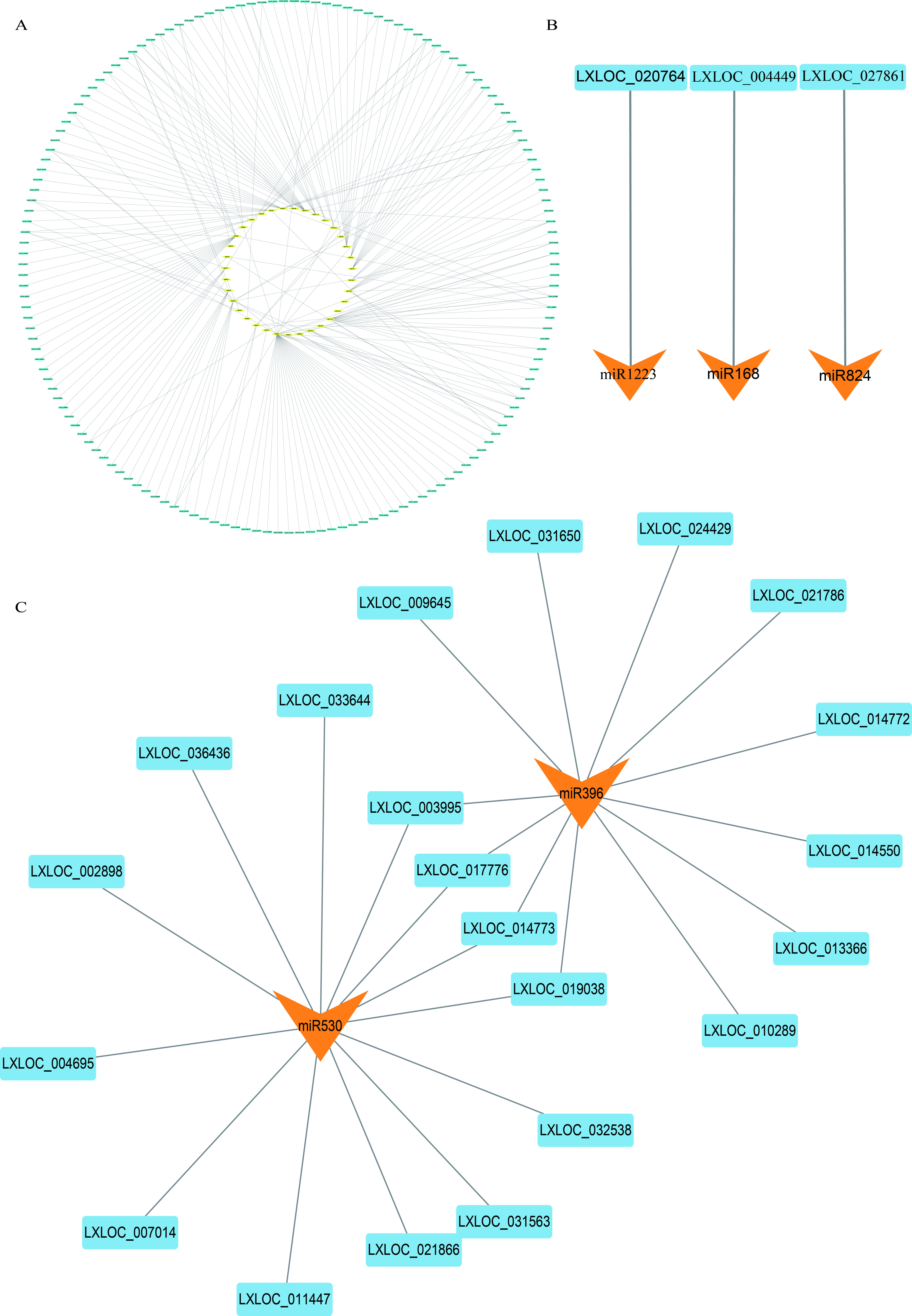

Supplement: Supplementary file 11 — Figure S4. Interaction networks of lncRNAs and miRNAs. The orange triangle indicates the miRNA families The blue rectangle indicates the related lncRNAs. The relationships among the lncRNAs and miRNAs are diverse. A) The lncRNAs and miRNA families have intersecting relationships. B) The relationships between with the lncRNAs and miRNA families are 1 to a model. C) The relationships between certain lncRNAs and miRNAs intersect. (JPG 4588 kb) [file 12863_2018_671_MOESM11_ESM.jpg]
